# Statistical Analysis of a Round-Robin Measurement Survey of Two Candidate Materials for a Seebeck Coefficient Standard Reference Material

**DOI:** 10.6028/jres.114.004

**Published:** 2009-02-01

**Authors:** Z. Q. J. Lu, N. D. Lowhorn, W. Wong-Ng, W. Zhang, E. L. Thomas, M. Otani, M. L. Green, T. N. Tran, C. Caylor, N. R. Dilley, A. Downey, B. Edwards, N. Elsner, S. Ghamaty, T. Hogan, Q. Jie, Q. Li, J. Martin, G. Nolas, H. Obara, J. Sharp, R. Venkatasubramanian, R. Willigan, J. Yang, T. Tritt

**Affiliations:** National Institute of Standards and Technology, Gaithersburg, MD 20899; Naval Surface Warfare Center, West Bethesda, MD 20817; RTI International, Research Triangle Park, NC 27709; Quantum Design, San Diego, CA 92121; Armor Holdings, Sterling Heights, MI 48310 and Michigan State University, East Lansing, MI 48824; Clemson University, Clemson, SC 29634; Hi-Z Technology, Inc., San Diego, CA 92126; Armor Holdings, Sterling Heights, MI 48310; Brookhaven National Laboratory, Upton, NY 11973; University of South Florida, Tampa, FL 33620; Advanced Institute of Science and Technology, Ibaraki, Japan; Marlow Industries, Inc., Dallas, TX 75238; RTI International, Research Triangle Park, NC 27709; United Technologies Research Center, East Hartford, CT 06108; General Motors R&D Center, Warren, MI 48090; Clemson University, Clemson, SC 29634

**Keywords:** bismuth telluride, consensus mean curve, Constantan, functional data analysis, Ridge regression modeling, round-robin, Seebeck coefficient, Standard Reference Material, thermoelectric

## Abstract

In an effort to develop a Standard Reference Material (SRM™) for Seebeck coefficient, we have conducted a round-robin measurement survey of two candidate materials—undoped Bi_2_Te_3_ and Constantan (55 % Cu and 45 % Ni alloy). Measurements were performed in two rounds by twelve laboratories involved in active thermoelectric research using a number of different commercial and custom-built measurement systems and techniques. In this paper we report the detailed statistical analyses on the interlaboratory measurement results and the statistical methodology for analysis of irregularly sampled measurement curves in the interlaboratory study setting. Based on these results, we have selected Bi_2_Te_3_ as the prototype standard material. Once available, this SRM will be useful for future interlaboratory data comparison and instrument calibrations.

## 1. Introduction

Thermoelectricity is the study of the direct conversion between thermal and electrical energy through the Seebeck and Peltier effects. In the Seebeck effect, a potential difference arises when a junction between two dissimilar conductors is heated or cooled [1].the Seebeck effect can be used for power generation applications. Conversely, when a current passes through the junction between two dissimilar conductors, heat is absorbed or expelled at the junction depending on the direction of current flow. This is known as the Peltier effect and can be used for electronic refrigeration [2].

Seebeck coefficient (*α*) is defined as the voltage (*V*) generated per degree of temperature difference between two points (*α* = *ΔV*/*ΔT*). The Seebeck effect has been used by NASA to supply power for deep space probes in its radioisotope thermoelectric generators (RTGs) and is of current interest to automobile manufacturers to supply additional power through waste heat recovery. RTGs have provided long term reliability with some deep space probes approaching three decades of constant operation. The Peltier effect can be used for electronics spot cooling of computer processors and has widely been used to thermally manage optoelectronic devices such as communication lasers and infra-red detectors. A more common use is in portable heaters/coolers that can be purchased inexpensively at many local stores. While wider use of thermoelectrics in more mainstream applications holds great promise because of their high reliability and environmental friendliness, the low efficiency with which they operate has restricted their usage. Recently, there has been a resurgence of activity in this field to find novel materials that can operate with higher efficiency to provide alternative power generation options and competition with conventional refrigeration technology.

The efficiency of a thermoelectric material is directly related to the thermoelectric figure of merit *ZT* given by *α*^2^*σT*/*κ* where *σ* is the electrical conductivity, *κ* is the thermal conductivity, and *T* is the absolute temperature. The current state of the art thermoelectric materials from the (Bi_1–X_Sb_X_) 2 (Te_1–Y_Se_Y_)_3_, Bi_1–X_Sb_X_, Si_1–X_Ge_X_, and PbTe systems all have maximum *ZT* values of around 1 at their respective optimum temperatures. Although this value has been the maximum for over 40 years, there exists no theoretical reason for this to be absolute limit [3]. Several recent reports have indicated that much higher *ZT*s are possible both in thin film superlattices [4] and in bulk materials [5]. A *ZT* of 3 to 4 would indicate an efficiency great enough to allow direct competition with conventional refrigeration devices [6]. While full evaluation of a material requires measurement of the electrical resistivity or conductivity, Seebeck coefficient and thermal conductivity, measurement of just the Seebeck coefficient can filter out those materials which do not have the desired thermoelectric properties. There exists a minimum Seebeck coefficient that must be achieved to give a desired *ZT*. If this Seebeck coefficient is not achieved, the material does not warrant further study as the other properties can not overcome a deficiency in the Seebeck coefficient. For *ZT* = 1, the Seebeck coefficient must be ≥ 157 μV/K; for *ZT* = 2, the Seebeck coefficient must be ≥ 222 μV/K. The derivation of this minimum Seebeck coefficient assumes the ideal case in which the lattice thermal conductivity is zero. Because the lattice thermal conductivity will not be zero in any real system, the actual Seebeck coefficient must be somewhat higher [7].

One of the needs that persist in this research field is that of a Seebeck coefficient standard reference material (SRM) to help ensure reliable measurements and characterization. Researchers building measurement equipment need to be able to calibrate their systems to known values in order to ensure consistency with different equipment in other laboratories. Numerous laboratories perform thermoelectric materials characterization through measurement of the electrical resistivity or conductivity, thermal conductivity, and Seebeck coefficient. These required measurements are demanding, especially the thermal conductivity measurements; however, one of the most important initial measurements is that of the Seebeck coefficient due to the minimum requirements. Standard reference materials exist for thermal conductivity and electrical conductivity, and there are reliable low Seebeck coefficient materials such as Pb or Pt; however, there is no high Seebeck coefficient SRM [8].

### 1.1 National Institute of Standards and Technology (NIST) and Thermoelectrics

Research efforts at NIST are guided by the NIST mission and vision statements. The NIST mission is “to promote U.S. innovation and industrial competitiveness by advancing measurement science, standards, and technology in ways that enhance economic security and improve quality of life.” The NIST vision is “to be the global leader in measurement and enabling technology, delivering outstanding value to the nation.”

With respect to the thermoelectric research community, the NIST mission and vision can be applied in two areas. First, NIST can help develop the metrology of thermoelectric measurements. A number of excellent thermoelectric measurement techniques are currently in use by the research community. However, these can be improved and new measurement techniques developed. Second, NIST can provide guidance and objectivity in measurements. This can be accomplished through development of standardized measurement procedures and methodologies, objective testing of results, uncertainty assessment, and development of standard reference materials.

The NIST Standard Reference Material (SRM) program currently offers over 1100 SRMs which are used for a variety of purposes such as instrument calibrations, accuracy verification, and new measurement techniques development. However, the program has not previousy looked at thermoelectric materials. As mentioned previously, full characterization of a thermoelectric material requires measurement of the Seebeck coefficient, electrical resistivity, and thermal conductivity, usually as a function of temperature. SRMs are currently available for the electrical resistivity and thermal conductivity. These are SRM 8420/8421 (electrolytic iron) and SRM 8424/8426 (graphite). Except for the electrical resistivity of graphite, the range of values covered by these SRMs is not typical of thermoelectric materials and hence not appropriate to calibration of measurement equipment used in the field. While these SRMs are not ideal, they do at least exist. There is no SRM for the Seebeck coefficient however. This is a void that needs to be filled as it is much needed by the thermoelectric research community.

### 1.2 Thermoelectric SRM Requirements

A number of aspects had to be considered when developing the Seebeck SRM. First, the material had to possess long-term stability. In addition, the material should be homogeneous and be able to be produced in a large consistent batch. This is because of the time and cost which would be required to individually certify each individual sample. Rather, a large homogeneous batch would allow for measurements of representative samples to provide data indicative of the whole batch. Second, the SRM had to be certified over a broad temperature range as most researchers in this field perform temperature dependent measurements. Measurements are usually divided into the low temperature regime (< 300 K) and high temperature regime (> 400 K). Thermoelectric research is active in both temperature regimes making SRMs needed for both. While there is normally some overlap between these regimes, they typically require different measurement equipment. Because of this, we determined that this SRM would be focused on one temperature regime. Third, it is important that the SRM possess a Seebeck coefficient that has magnitude on the order of that typically measured in the field. These values should be somewhere from 25 μV/K to 400 μV/K. Somewhere in the middle of this range would be ideal. Fourth, the SRM should be available at a reasonable price to the community; therefore the development and production must be cost-effective. Also, there should be sufficient demand for the SRM which in turn has an impact on the price. Fifth, as we consider development of the SRM, some thought must be given to future SRMs. It might be possible to use the same material for future thermoelectric-related SRMs if chosen properly. Future SRMs could be produced over a broader or different temperature range, for different properties or for *ZT*, or for other sample geometries such as thin film.

## 2. Round-Robin Measurement Survey^1^

We initiated a measurement survey to determine the feasibility of producing the SRM, the consistency of the candidate materials, and the best measurement technique for providing the standard data. Two candidate materials were chosen. Constantan is well known as a simple alloy (55 % Cu/45 % Ni) commonly used in thermocouples with a moderate Seebeck coefficient at room temperature. Cylindrical samples (6.47 mm long by 3.45 mm diameter) were purchased from Concept Alloys. Bi_2_Te_3_ is a state of the art thermoelectric material with a high Seebeck coefficient at room temperature. Undoped samples were obtained from Marlow Industries in a rectangular shape (6.08 mm long by 3.04 mm square).

Although standards are needed in both the low and high temperature regimes, for this SRM we decided to focus on the low temperature range from 10 K to 390 K. This decision was made because of previous experimental experience in this temperature regime and the availability of measurement equipment. While this standard primarily provides data for the low temperature regime, it will also provide some overlap with the low end of high temperature equipment until a standard can be provided for those temperatures.

A number of laboratories were enlisted to participate in this survey. These are a mixture of laboratories involved actively in thermoelectric research and represent industry, university, and government laboratories both domestic and international. These participants and the primary researcher from each are listed in Table 1.

### 2.1 Measurement Equipment

A number of measurement systems were used in this study including both commercial and custom-built systems. The measurements were carried out with several different measurement techniques (some systems were capable of multiple techniques).

#### 2.1.1 Commercial Systems

The Quantum Design Physical Property Measurement System (PPMS) with Thermal Transport Option (TTO) is a versatile system which can measure the Seebeck coefficient from 2 K to 400 K in several different modes, each of which was used in this study. Samples can be mounted in either a 2 or 4-probe configuration, and measurements can be performed with a stable sample temperature or dynamic sample temperature (usually ≤ 0.5 K/min). The dynamic measurements continuously monitor the *ΔT* and *ΔV* along the sample while supplying a heat pulse to one end and slowly varying the sample temperature. This approach gives the ability to measure the Seebeck coefficient as a function of temperature without having to wait for stability and data collection at each temperature. The steady-state values for *ΔT* and *ΔV* are found by extrapolating the data from a relatively short heat pulse. This system prefers a sample geometry such that the thermal conductance at 300 K is between 1–5 mW/K for 2-probe measurements. Bar- or disc-shaped, gold-plated, copper contact leads were used and attached to the sample with either solder or silver epoxy (EpoTek H20E). The versatility of this system also allows for integrating 3^rd^ party electronics and/or software to perform custom measurements. One laboratory provided data using this system with a Keithley nanovoltmeter to measure the Seebeck voltage while performing a direct steady-state DC measurement.

The ULVAC RIKO ZEM-2 system performs a steady-state sweep technique and operates in two modes to cover different temperature regimes. The cryostat mode allows measurements from 193 K - 373 K while the furnace mode allows measurements from room temperature to 1273 K. This system prefers samples 13 mm or longer while at least 8 mm of length is recommended by the vendor. Using samples shorter than this length introduces error due to smaller probe spacing and temperature difference. The samples in this study were only 6 mm long and required extenders to span the length not covered by the sample. A 4-probe measurement geometry was used with chromel or platinum lead wires attached to the ends of the samples and Type K (Type M8 and L) or R(Type M10) thermocouple probes attached to the sides. In this steady-state sweep technique, the sample was held at a constant temperature while one end of the sample was heated to produce a constant temperature gradient. The temperature and voltage difference between the thermocouple probes was measured. The next temperature diference value was attained, and measurements were repeated. After all temperature difference setpoints at a particular sample temperature were covered, the slope of the voltage difference (*ΔV*) vs temperature difference (*ΔT*) gave the Seebeck coefficient at that sample temperature. After this, the sample temperature was changed, and the measurement was repeated.

#### 2.1.2 Custom Systems

Three laboratories used systems which allowed for measurements over a broad temperature range covering much of the target range for this study. Each of these employed different measurement techniques and sample mounting, however.

The first system used a steady-state sweep technique in which the sample was held at a constant temperature and the *ΔT* was slowly ramped through a range of values while monitoring the *ΔV*. The data was linearly fit, and the slope yielded the Seebeck coefficient. A small resistor was epoxied to the top of the sample, and the opposite end was soldered to a heat sink. Two differential thermocouple contacts were made to the sides of the sample for measuring the *ΔT*, and a thermocouple epoxied between the differential thermocouple contacts measured the average sample temperature.

The second system used a 4-probe configuration in which current was pulsed through a small platinum heater resistor on one end of the sample to generate the *ΔT*. The other end of the sample was attached to the probe using solder or silver paste. Silver paste was used to attach type-E thermocouples to the sample to measure the *ΔT*.

The third system used a pseudo-steady-state technique in which a constant *ΔT* was applied along the sample, and measurements of the *ΔV* were made as the sample temperature was slowly changed (≤ 1 K/min). A smaller *ΔT* calculated from a percentage of the sample temperature was used as the temperature was decreased. Samples were soldered between 2 copper blocks which acted as voltage probes for measuring the *ΔV*. The junctions of a differential thermocouple were embedded in the copper blocks to measure the *ΔT*.

The other systems only measured at or near room temperature. Three of these used a simple *ΔT* sweep technique but had sight sample mounting variations. In the first technique, copper end caps were soldered to the ends of the sample, and each cap included a copper wire and a 3 mil Type T thermocouple. One end of the system was thermally sunk to a thermoelectric cooler to provide basic sample temperature control. In the second technique, samples were mounted between 2 copper blocks and partially exposed above the blocks. To the exposed parts, voltage and thermocouple probes were attached. Cartridge heaters were embedded in each block to control the *ΔT*. Two measurements were performed at each temperature with reversed thermocouples to account for thermocouple variations. The sample was slowly swept through a range of *ΔT* values which centered on the temperature being measured. In the third technique, samples were clamped between two clean copper blocks each embedded with a heater and thermocouple. The blocks were held at different temperatures and ramped slowly through different *ΔT* values while the *ΔV* was recorded. A linear fit to the data gave the Seebeck coefficient.

One of the other systems used a basic single point measurement. Samples were mounted between 2 nickel-plated copper blocks held at different temperatures to produce a *ΔT* along the sample. The *ΔV* between the 2 blocks was measured and divided by the *ΔT* to give the Seebeck coefficient.

The last system used a Harman technique in which a *ΔT* was produced along the sample by means of the Peltier effect when a current was passed through the sample. After stabilization, the current was switched off; and the ohmic and Seebeck voltages were separated from the total voltage. Measurements were repeated using opposite current sense to account for thermocouple differences and voltmeter offsets.

### 2.2 Round-Robin Procedure

The measurements were conducted in two rounds to allow each sample to be measured by 2 different laboratories and provide a good amount of comparative data while working within the time constraints of the project and the participants. The ideal situation would be where each sample is measured by all laboratories. However, due to the nature of these measurements, this would require an extreme time commitment by each laboratory and would greatly lengthen the SRM project as a whole. This was not practical. The procedure we used allowed each measurement technique to be performed on 2 different samples and for each sample to be measured by 2 different laboratories. Also, multiple samples were measured at NIST using one technique to provide additional sample consistency data.

Two samples of each candidate material were sent to each laboratory. One sample of each was to be measured while the other served as a backup. Some laboratories provided data on both samples. Each laboratory was asked to perform a minimum of 2 measurements on each sample and more if necessary to provide confidence in the final data. Also, each laboratory was asked to use their normal techniques and multiple techniques if available and if time allowed.

The measured samples were then sent back to NIST where they were randomly assigned to a different laboratory for the second round of measurements. Other switching arrangements were discussed and considered at length. We considered hand selecting some of the switching to insure certain comparisons would be made between specific laboratories and their measurement techniques. In the end, however, it was decided it would be better to allow switching to be fully random so that the broadest number of comparisons would be possible. The samples were then sent out to the laboratories again for the second round of measurements.

## 3. Measurement Data and Parametric Representation

**Fig. 1:**
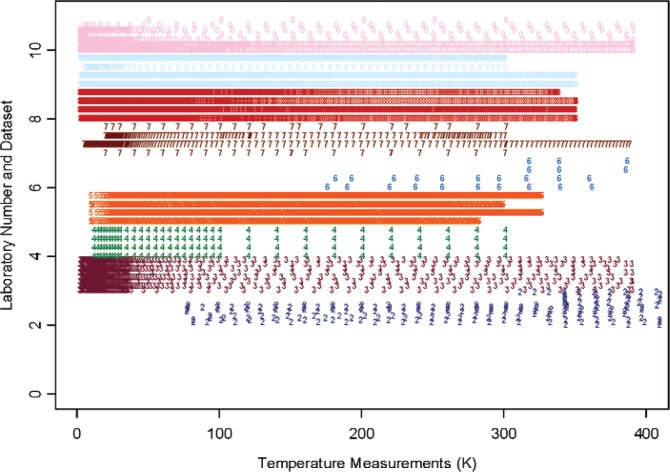
Density of temperature measurement data for material Constantan. The y-axis represents the numerical labels assigned to the 9 out of 12 laboratories as shown in Table 1, and the decimal points represent different datasets from the given laboratory. The temperature unit is Kelvin (K). The same color and numeric label are used for all data from each particular laboratory.

**Fig. 2:**
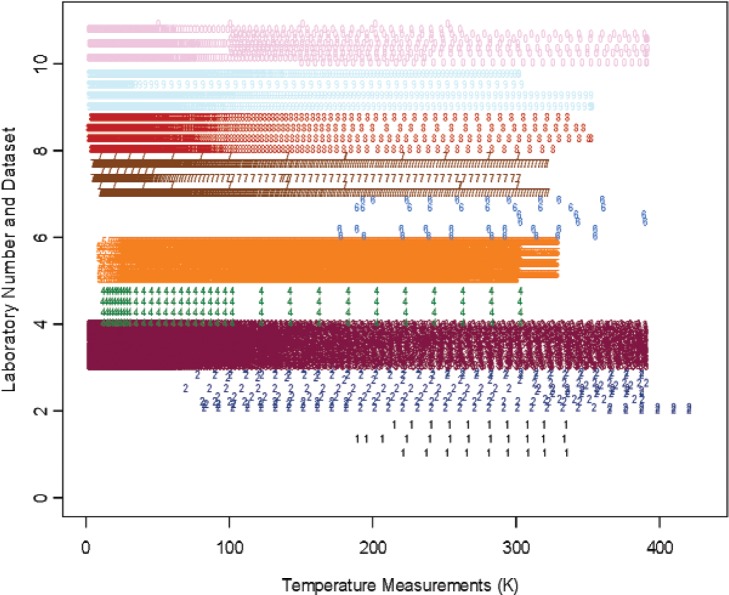
Density of temperature measurement data for material Bi_2_Te_3_. The *y*-axis represents the numerical labels assigned to the 10 out of 12 laboratories as shown in Table 1, and the decimal points represent different datasets from the given laboratory. The temperature unit is Kelvin (K). The same color and numeric label are used for all data from each particular laboratory.

There are issues which present difficulty when analyzing and combining measurement data curves from different measurements, laboratories, or techniques. First, the data covers different temperature ranges with different numbers of sampling points or data density. We assign numerical labels 1, 2, 3, 4, 5, 6, 7, 8, 9, 10 for the 10 laboratories whose data are accepted, and we use decimal points within each interval to represent the different datasets from a particular laboratory. The temperature sampling points for all measurement data are shown in Fig. 1 for Constantan and Fig. 2 for Bi_2_Te_3_. Each color/numeric label represents all the data from a particular laboratory. It is seen that the temperature range and density of each measurement data set differs greatly between laboratories, and even within the same laboratory. These variations cause difficulty when comparing and combining the different measurements. We use a parametric model for the measurement curves in order to interpolate and to analyze multiple curves.

### 3.1 Parametric Interpolating Model

In order to analyze the variability in the irregularly and sparsely sampled measurement data, we first entertain data representation through parametric models via multiple regression analysis [9]. We imagine each individual measurement data set from one of the *m* laboratories consists of

$$\begin{array}{l} y_{ij}(t_{ijk})=f_{ij}(t_{ijk})+e_{ij}(t_{ijk}),\\ i=1,\ldots m,j=1,\ldots n_{i};k=1,\ldots s_{ij} \end{array}$$ (1)

where *y_ij_* (*t_ijk_*) denotes the measurements at temperature points *t_ijk_* by the *j*th measurement set within the *i*th laboratory, and *f*_0_ (*t_ik_*) is the common (true) curve evaluated at *t_ijk_*. The measurement errors (including interpolation, laboratory, and sample variability, etc.) and lack of fit error due to the use of a parametric model are summarized by the residual error term *e_ij_* (*t_ijk_*) which is assumed to have a normal distribution *N*(0, *σ_ij_*^2^(*t_ijk_*)) where *σ_ij_*^2^(*t_ijk_*) should include the parametric model error for the *j*th measurement of the *i*th laboratory. We use a parametric model for *f_ij_* (*t_ijk_*). The purpose of the model is to adequately approximate the data with a parametric form; there is no physical meaning associated with the parameters. The benefit is to have a set of finite-dimensional parameters as a proxy summary of individual measurement curves.

Applying (1), we identified a multiple linear regression model [10] which seemed to fit the available measured data set very well (see also comments in Sec. 6).

$$\begin{array}{l} y_{ij}\left(t_{ijk}\right)=a_{ij0}+a_{ij1}\log\left(t_{ijk}+1\right)+a_{ij2}\sqrt{t_{ijk}}\\ \;+a_{ij3}\sin\left(\frac{2\pi t_{ijk}}{700}\right)+a_{ij4}\cos\left(\frac{2\pi t_{ijk}}{700}\right)+e_{ij}\left(t_{ijk}\right) \end{array}$$ (2)

where *y_ij_* (*t_ijk_*) is the measured Seebeck coefficient (μV/K) at temperature *t_ijk_* (Kelvin). The vector **a***_ij_* = (*a_ij_*_0_, *a_ij_*_1_, *a_ij_*_2_, *a_ij_*_3_, *a_ij_*_4_)*^T^* represents the parameterization of the measured curve.

### 3.2 Parameter Estimation

To estimate the parameters in Eq. (2) for each data set, the standard least squares method used in our earlier work [10] can be improved due to the instability in the least squares estimator when the measured temperature points are few or limited in a small range. Let *X* denote the *n* × *p* design matrix consisting of 5 columns defined by the regression terms in (2) and rows which are evaluated at each sampling point. Let *Y* denote the Seebeck coefficient response vector. The least squares estimator is given by

$$\hat{\beta }={\left(X^{T}X\right)}^{-1}X^{T}Y;\;\hat{Y}=X\hat{\beta }.$$ (3)

The problem with the standard least squares method applying to (2) is that *X^T^X* is near singular when the sample size is small or the temperature measurement range is narrow. As a consequence, the estimated parameters can be highly variable and unstable; and the uncertainties associated with the estimated parameters are extremely large. To alleviate the problem one can use the Ridge regression method [11] by introduction of smoothing parameter *k* to stabilize the inverse computation given by

$${\hat{\beta }}_{R}={\left(X^{T}X+kI\right)}^{-1}X^{T}Y;\;{\hat{Y}}_{R}=X{\hat{\beta }}_{R}.$$ (4)

If we denote the singular value decomposition of *X* by *X* = *UDV^T^*, then *X^T^X* = *VD*^2^*V^T^*,

$$\begin{array}{l} {\hat{\beta }}_{R}={\left(VD^{2}V^{T}+kI\right)}^{-1}VDU^{T}Y=V{\left(D^{2}+kI\right)}^{-1}DU^{T}Y\\ \;=\sum_{i=1}^{p}\left(\frac{\delta_{i}}{\delta_{i}^{2}+k}\right)\left(u_{i}^{T}Y\right)V_{i} \end{array}$$ (5)

where *D=diag*{*δ*_1_,…, *δ_p_*},U=(*u*_1_,…,*u_p_*), *V* = (*V*_1_,…,*V_p_*) and

$${\hat{Y}}_{R}=UD{\left(D^{2}+kI\right)}^{-1}DU^{T}Y=\sum_{i=1}^{p}\left(\frac{\delta_{i}^{2}}{\delta_{i}^{2}+k}\right)\left(u_{i}^{T}Y\right)u_{i}.$$ (6)

Also, if we denote *A*(*k*) = *UD*(*D*^2^ + *kI*)^−1^ *DU^T^*, then *Y_R_* = *A*(*k*)*Y*.

The choice of *k* requires careful considerations. A large *k* reduces the variance in the resulting estimator while incurring potentially large bias. We try to select *k* that gives a stable estimator and has negligible bias. A formal procedure for choosing *k* is based on the Generalized Cross-validation criterion [12] by minimizing the prediction variance

$$\begin{array}{l} \Omega (k)=\frac{\frac{1}{n}{\left\|\left(I-A(k)Y\right)\right\|}^{2}}{{\left[\frac{1}{n}tr(I-A(k))\right]}^{2}}\\ \;=\frac{\frac{1}{n}\left[\sum_{i=1}^{n}y_{i}^{2}-\sum_{i=1}^{p}\frac{\delta_{i}^{2}\left(\delta_{i}^{2}+2k\right)}{{\left(\delta_{i}^{2}+k\right)}^{2}}\left(u_{i}^{T}Y\right)\right]}{{\left[1-\frac{p}{n}+\frac{1}{n}\frac{k}{\delta_{i}^{2}+k}\right]}^{2}}. \end{array}$$ (7)

In practice, we find that the smallest *k* among the feasible values is always preferred. This indicates that our chosen estimators are close to those given by using the generalized inverses. If we let *X*^+^ denote the Moore-Penrose inverse of a matrix *X*, then *X*^+^ = (*X^T^X*)^+^*X^T^*; and *X*^+^ satisfies the following conditions [13]:

$$X^{+}X,XX^{+}\;\text{are symmetric}$$ (8)

$$XX^{+}X=X,X^{+}XX^{+}=X^{+}.$$ (9)

If *X*= *UDV^T^*, then *X*^+^= *VD ^+^U^T^* where *D^+^* is the transpose of *D* whose positive singular values are replaced by their reciprocals. When *k* ***→*** 0, the Ridge regression estimator in (4) converges to the Moore-Penrose generalized inverse estimator given by:

$${\hat{\beta }}_{+}=X^{+}Y={\left(X^{T}X\right)}^{+}X^{T}Y.$$ (10)

The estimator is a least squares solution to the following problem: its norm ‖*β*‖_2_ is minimized among all vectors *β* for which

$${\left\|Y-X\beta \right\|}_{2}$$ (11)

is minimized. The corresponding fitted regression line is given by

$${\hat{Y}}_{+}=X{\left(X^{T}X\right)}^{+}X^{T}Y=XX^{+}Y.$$ (12)

The covariance of ${\hat{\beta }}_{+}$ is given by

$$Cov({\hat{\beta }}_{+})=\sigma^{2}{\left(X^{T}X\right)}^{+}$$ (13)

where we assume *Cov* (*Y*) = *σ*^2^*I*. Note that the Ridge regression estimator may be biased. A useful notion is estimable function (or linear combination of parameters) for which there exists unbiased estimate based on linear combination of data. This is the essence of the theory of the Gauss-Markov model and for estimable functions there are simplifying expressions for uncertainty analysis [14].

The adequacy and validity of the parametric model as an approximate representation of the measurement data curves can be checked via comparison to the non-parametric model results using the locally weighted regression (LOWESS), which is available in S-plus^2^ and other statistical softwares [15, 16].

**Fig. 3:**
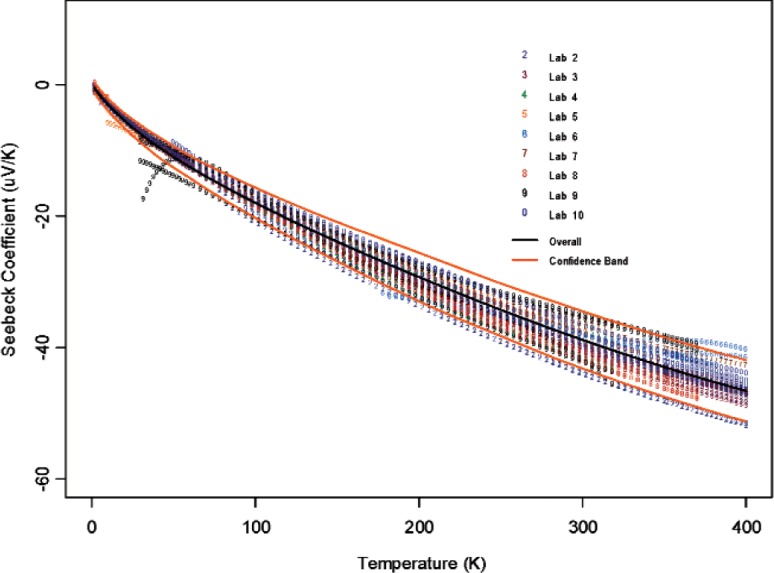
Fitted measurement curves by laboratory on the Constantan material.

**Fig. 4:**
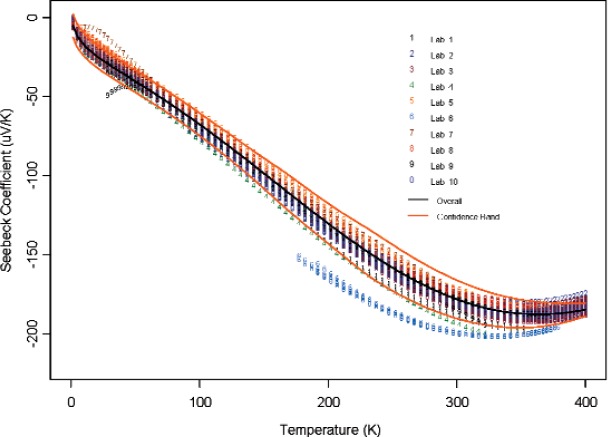
Fitted measurement curves by laboratory on the Bi_2_Te_3_ material.

If we accept that Eq. (2) provides an adequate representation of measurement data curves across different samples and laboratories, see Fig. 3 and Fig. 4, the question arises as how much meaning can be attached to the parameters and how much the variability in parameter estimates can account for the measurement variability across samples or laboratories. Two measurement data curves may have different representation with vastly different coefficients due to the difference in measurement data range and due to instability from under-sampling and over-parameterization within the data range. The data range is likely the result of different measurement equipment used. When the number of sampling points is small or when the measured data points do not support the complexity of the presumed model, the Ridge regression approach becomes a preferred one to use over the standard least squares method. The lack of parameter identifiability or *parameter redundancy* is a well-known problem in nonlinear regression [17, 18] and can be caused by the intrinsic nature of parameterization in nonlinear representations. Because of this, our view is to use the parametric representation as an interpolation tool only; and it appears that the fitted parameters do not have much use beyond this data summarization stage.

## 4. Meta Analysis: Combining Irregularly Sampled Curves

### 4.1 Consensus Mean Curve

After we have summarized the irregularly sampled measurement data curves through a parametric model, all data among the samples and laboratories can be compared on the measured data points or through interpolations via the parametric fits. The first important issue is to define the consensus mean curve for a particular group of measurement curves. The naïve approach is to use the mean of the fitted regression coefficients which we call the “mean regression” approach, in which the regression coefficients from each measurement curve are weighted equally. This approach does not work well due to vast variability in the parameter estimates. The second approach is to fit a single model to all data from that group which we call the “all data regression” approach. We see that “all data regression” approach appears to give consistently the most sensible results. This approach is equivalent to the weighted vector mean approach in which the regression coefficient vectors are weighted according to the inverse of the least squares covariance matrices, Eq. (13) [19]. However, we caution the readers that the regression coefficient vectors are too heterogenous to be analyzed using standard statistical procedures such as meta analysis as those mentioned in the comprehensive review by Becker and Wu [20]. The reasons are that, in addition to huge differences in measurement uncertainty in some measurement curves due to limited sampling points, there are significant differences in measurement data ranges, and there are substantial between-laboratory differences in the measured temperature points. All these make the resulting regression coefficients less comparable, and make direct analysis based on the fitted regression coefficients very difficult. We argue that the regression coefficients should be treated as a function of data range as well as sample size and estimation uncertainty. To avoid the complications, datasets which have less than 5 data points in the focus range were not considered, since the fitted model were completely unreliable or the data were considered unreliable by the contributing laboratory. This resulted in 55 datasets being used for Constantan and 114 data sets being used for Bi_2_Te_3_. Thus, when we are comparing and evaluating the variability of the measurement curves, we focus on the interpolated measurement curves based on the fitted regression functions and use interpolated values when there are no direct measurement data.

### 4.2 Smooth Variance Estimation and Confidence Intervals

Another problem associated with the statistical analysis of the round robin data is the development of a confidence band for the consensus mean curve *m*(*t*). We find that the most sensible approach is to first compute the curves at the desired range using the coefficients of the parametric model fitted to each data from each laboratory, and then compute the pointwise variance *v*(*t*)as the mean of the squares of deviations of each curve from the central curve *m*(*t*). The pointwise estimated functional variance may be very rough, and it can be smoothed using LOWESS with a small bandwidth (e.g., we use f = 0.2, 20 % of local data points in the local fitting). To compute the confidence band, we simply use $m(t)\pm c\sqrt{v(t)}$ with *c* = 2 which gives the pointwise 95% confidence intervals (if the uncertainty in the variance estimate can be ignored). There is an interesting interpretation of the pointwise confidence intervals: if one treats the two confidence bands as two boundary lines, and calls any measured or interpolated values on a curve lying outside the two bounds the exceedances points, then the percentage of the exceedances as a fraction of the total temperature points summed over all measurement curves tends to 5 %, so asympototically the confidence intervals have the desired average spatial coverage probability of 95 %. Similar notion of confidence intervals is discussed by Wahba [21] who also coined the name of Bayesian confidence intervals, and by Nychka [22] who proved that the pointwise confidence intervals in the context of a smoothing spline regression has the required specified average coverage probability.

## 5. Statistical Analysis Results

Using the “all data regression” approach and Eq. (2), we modeled all data for the 2 candidate materials which gave

$$\begin{array}{l} y_{1}(x)=-0.09+1.81\log(x+1)-2.79\sqrt{x}\\ \;+0.93\sin\left(\frac{2\pi x}{700}\right)+1.39\cos\left(\frac{2\pi x}{700}\right); \end{array}$$ (14)

for Constantan and

$$\begin{array}{l} y_{1}(x)=-55.10-4.79\log(x+1)-2.49\sqrt{x}\\ \;-1.88\sin\left(\frac{2\pi x}{700}\right)+57.61\cos\left(\frac{2\pi x}{700}\right) \end{array}$$ (15)

for Bi_2_Te_3_. These results are plotted in Figs. 3 and 4 respectively with all the data used for the model.

**Fig. 5:**
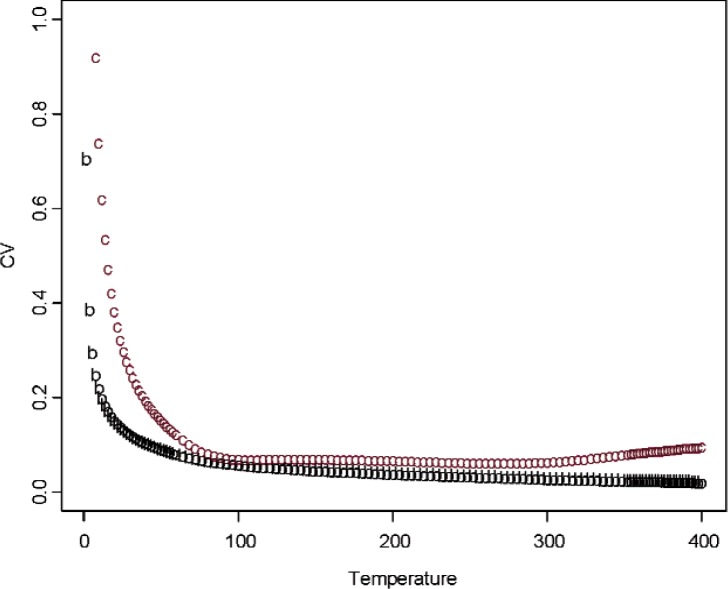
Sample-to-sample measurement uncertainty as a fraction of absolute consensus mean signal (“b” for Bi_2_Te_3_; “c” for Constantan).

The variability among the measurement curves is quantified through variance function defined as the mean of the squares of the deviations of each curve from the central curve. The variance function can be very rough at some tempearure range and it is smoothed out via the LOWESS smoothing function. The coefficient of variation (CV) at each temperature point is computed as the standard deviation divided by the absolute consensus mean value. The CVs as a function of temperature for both Constantan and Bi_2_Te_3_ when the variance function is computed over all the measurement curves of samples are plotted in Fig. 5. The standard deviation for Constantan data is increasing as a function of temperature, and CV is nearly constant for temperature above 100 K. For Bi_2_Te_3_, the standard deviation is nearly constant across temperature. It is seen that the CV for Bi_2_Te_3_ is smaller than the CV for Constantan. Based on the results of our data analysis, the fact that Bi_2_Te_3_ has a larger absolute Seebeck coefficient value, and also most laboratories have measurement techniques for the Bi_2_Te_3_ at a wide range of temperature values that we are interested in, we have selected Bi_2_Te_3_ as our candidate Standard Reference Material (see Sec. 6 for more discussion). Besides, Bi_2_Te_3_ is currently one of the materials being used by industry for cooling applications.

**Fig. 6:**
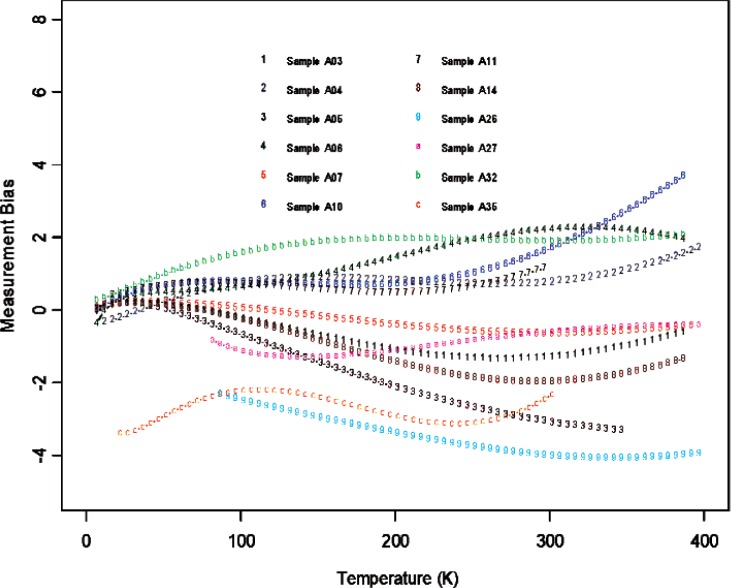
Sample bias (deviations from the consensus mean curve, with potential laboratory and technique differences) for all the samples used in the studies for Constantan.

**Fig. 7:**
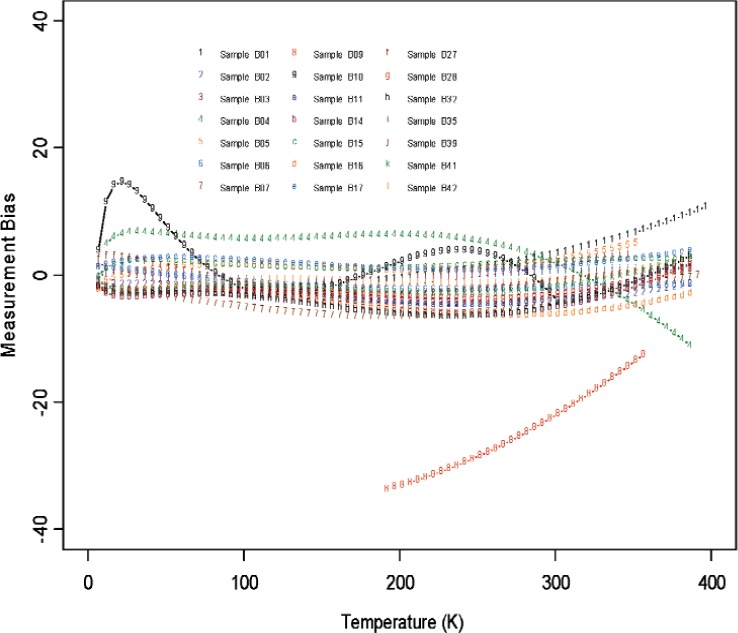
Sample bias (deviations from the consensus mean curve, with potential laboratory and technique differences) for all the samples used in the studies for Bi_2_Te_3_.

**Fig. 9:**
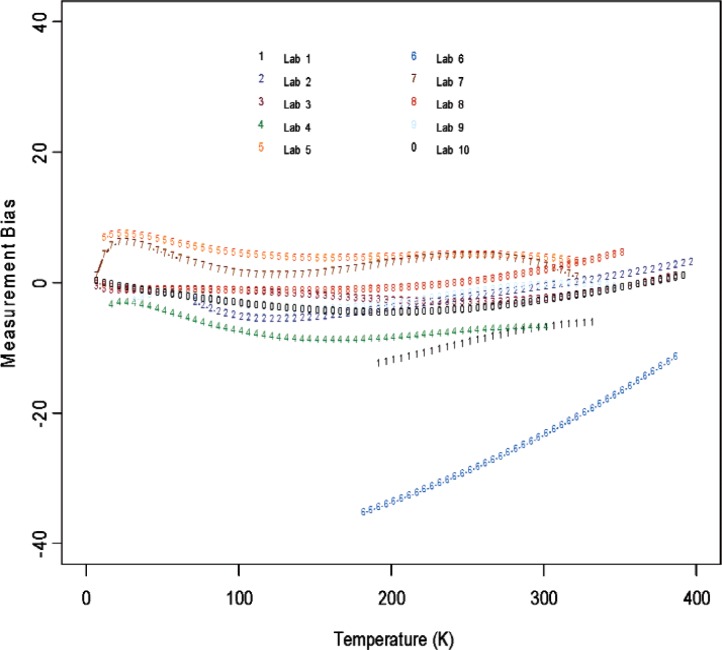
Laboratory bias (deviations from the consensus mean curve, with potential sample and technique differences) for Bi_2_Te_3_.

**Fig. 11:**
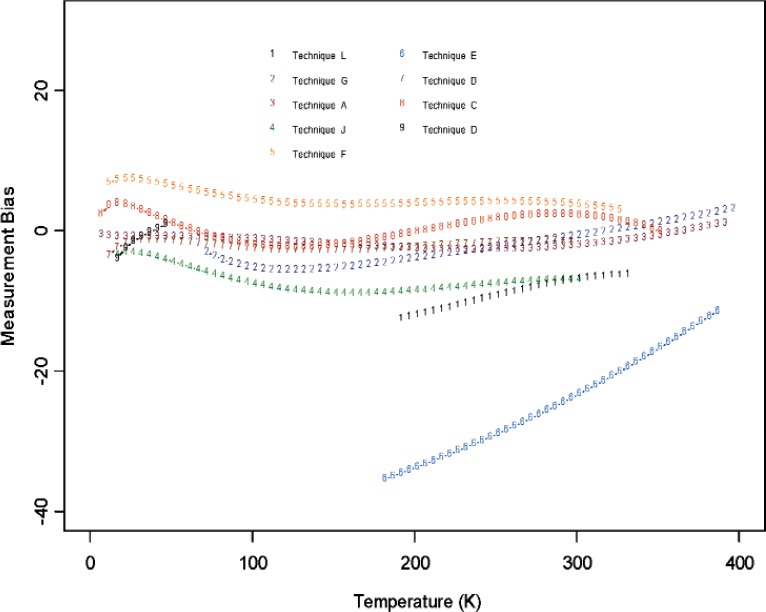
Measurement technique bias (deviations from the consensus mean curve, with potential laboratory and sample differences) used in the studies for Bi_2_Te_3_.

From Fig. 6 through Fig. 11, we report the deviations from the consensus mean curve due to three factors (Sample, Laboratory, or Measurement Technique) that may affect measurement performance for each of the two materials, Constantan and Bi_2_Te_3_. The samples were assigned randomly in the first round and then switched to another laboratory in round two, so there are typically two or more samples being measured by each laboratory. Each laboratory was asked to use their most reliable measurement technique, and some laboratories may have used up to four techniques for measurements. In this very exploratory experimental design set we do not apply rigorous statistical design involving orthogonality in order to separate the effect of measurement techniques from the laboratory, therefore the effect of laboratory is strongly coupled with the techniques being used. The confounding effect with choice of samples is less of an issue since there were enough samples being measured and samples were usually measured twice by two different laboratories. The outlying measurements seen in Fig. 4 from a single laboratory (Lab 6) show up also in Figs. 7, 9, and 11. We believe this is caused by a single laboratory using measurement technique E, the reasons being that some of the same samples have been measured by another laboratory without producing the pronouced deviations. Overall, we consider our interlaboratory study to be successful in achieving good agreements in measurements from the volunteering participating laboratories and in the identification of reliable measurement techniques in the desired wide temperature range which we are interested in pursuing.

## 6. Summary

To summarize, our procedure for statistical analysis of irregularly sampled measurement curves in the inter-laboratory study consisted of the following steps.

1. Each measurement data is fitted to a parametric model Eq. (2). The tuning parameter choice in the Ridge regression parameter estimation and goodness of fit are checked through the nonparametric LOWESS models. We arrive at a parametric representation of each measurement curve; and at every temperature point within the measurement range, the Seebeck coefficient can be computed based on the fitted model.
2. For measurement performance comparison, whether it is sample-to-sample, laboratory-to-laboratory, or technique-to-technique, at a given common set of temperature values, we compute the predicted Seebeck values on the common temperature points based on the fitted parametric model, and then compute the standard deviation at each temperature point.
3. The common mean for multiple measurements is given by fitting the parametric model (2) to all the combined data.
4. The final confidence band is given by the common mean plus or minus the stanadard deviation multipled by the coverage factor *k* = 2, which gives the 95 % average coverage probability assuming the normal distribution. The bias of each measurement is computed as the difference between the computed measurement point from the fitted parametric model and that from the common mean model.

Our study offers a few lessons which may be beneficial for future design and analysis of interlaboratory experiments involving sampled curves and functions. The significant differences (cf. Fig. 1 and Fig. 2) in the sampling design from different laboratories and different replicates have made analysis based on the parameters of an interpolating model unsuitable. We emphasize that the proposed model (2) is just one of the many interpolating models that can be used. For example, we have recently discovered another model in our latest Seebeck coefficient SRM work,

$$\begin{array}{l} m(t)=a_{0}+a_{1}t+a_{2}{\left(t-200\right)}^{2}\\ \;+a_{3}{\left(t-200\right)}^{3}+a_{4}{\left(t-200\right)}^{4} \end{array}$$ (16)

which also fits the round robin data well. However, we should point out that fitting of this model to the round robin data still presents the same challenges as the linear terms cannot be reformulated into orthogonal terms because of the vast differences in the sampling design of each data set, and orthogonality depends on the design of data sets. The strong multicollearity in the less sampled data set makes the use of Ridge regression necessary, though it is more difficult to compare the different data sets based on the fitted parameters. That is the reason why we emphasize that the parametric model has served our purpose of interpolation within each data set very well, but the fitted parameters have no physical meanings and have vast variations across different data sets. Another important lesson is that, we have not enforced a good statistical design so that the confounding effect of measurement technique and laboratory effects may be reduced. In the future when there are more laboratories who can use multiple techniques, a good choice of experimental design may become feasible.

Based on the results of the round-robin measurement survey, Bi_2_Te_3_ will be used for the SRM. To this end, 400 units have been purchased from Marlow Industries with sample dimensions of 8 mm × 3.5 mm × 2.5 mm. This sample has different dimensions than those used for the round-robin measurement survey based on feedback from the participants. These dimensions allow more room for 4-probe resistivity measurements while maintaining an appropriate thermal conductance.

Bi_2_Te_3_ will be certified as the SRM at NIST with the standard data produced using a Quantum Design Physical Property Measurement System with some modifications including 3^rd^ party electronics and custom software. The details of this system and technique will be discussed elsewhere.

## Figures and Tables

**Fig. 8:**
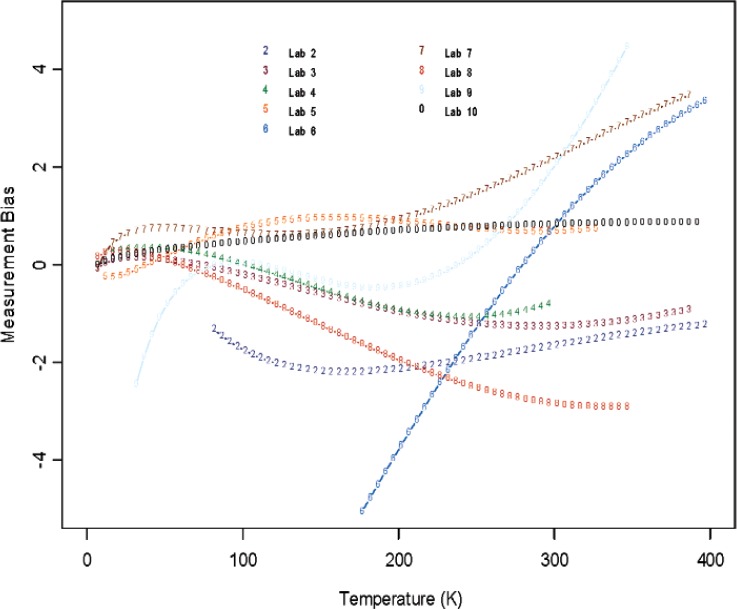
Laboratory bias (deviations from the consensus mean curve, with potential sample and technique differences) for Constantan.

**Fig. 10:**
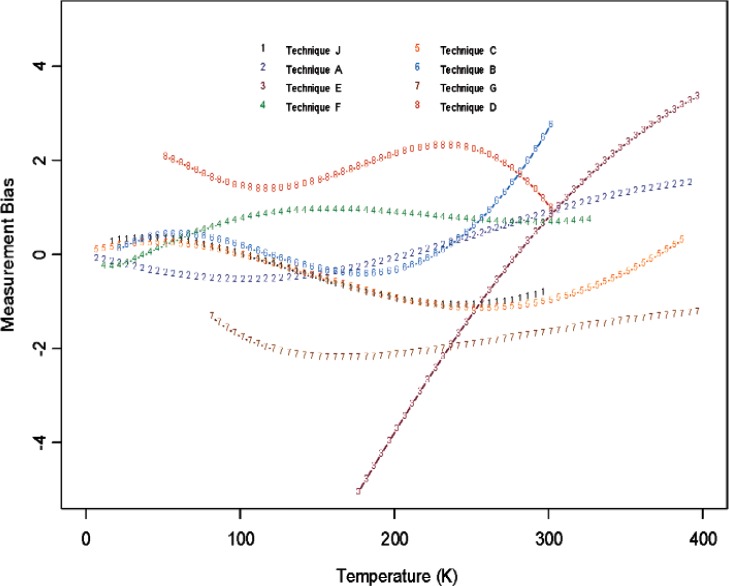
Measurement technique bias (deviations from the consensus mean curve, with potential laboratory and sample differences) used in the studies for Constantan.

**Table 1:** Round-robin measurement survey participants

Primary Researcher	Laboratory
Neil Dilley	Quantum Design
Norbert Elsner	Hi-Z Technology
Tim Hogan	Michigan State University
Qiang Li	Brookhaven National Laboratory
Nathan Lowhorn	National Institute of Standards and Technology
George Nolas	University of South Florida
Haruhiko Obara	National Institute of Advanced Industrial Science and Technology—Japan
Jeffrey Sharp	Marlow Industries
Terry Tritt	Clemson University
Rama Venkatasubramanian	RTI International
Rhonda Willigan	United Technologies
Jihui Yang	General Motors

## Footnotes

1 The purpose of identifying the equipment in this article is to specify the experimental procedure. Such identification does not imply recommendation or endorsement by the National Institute of Standards and Technology.

2 S-plus is a trademark of Insightful Corporation. Mention of a software product in this paper is only to illustrate and to make explicit the statistical procedures used in our data analysis, and does not imply in anyway the endorsements of NIST.
